# Peripheral Arterial Stiffness in Acute Pulmonary Embolism and Pulmonary Hypertension at Short-Term Follow-Up

**DOI:** 10.3390/jcm10143008

**Published:** 2021-07-06

**Authors:** Silvia Papa, Cristiano Miotti, Giovanna Manzi, Gianmarco Scoccia, Federico Luongo, Federica Toto, Claudia Malerba, Nadia Cedrone, Elena Sofia Canuti, Annalisa Caputo, Giulia Manguso, Serena Valentini, Susanna Sciomer, Francesco Ciciarello, Giulia Benedetti, Francesco Fedele, Carmine Dario Vizza, Roberto Badagliacca

**Affiliations:** 1 Department of Clinical, Internal Medicine, Anesthesiology and Cardiovascular Sciences, Sapienza University of Rome, 00161 Rome, Italy; silviapapa83@gmail.com (S.P.); miotticristiano@gmail.com (C.M.); giovannamanzi91@gmail.com (G.M.); gianmarcoscoccia@gmail.com (G.S.); federico.luongo91@gmail.com (F.L.); federica.toto@uniroma1.it (F.T.); claudiamalerba4@gmail.com (C.M.); 05escanuti@gmail.com (E.S.C.); annalisa.caputo1995@gmail.com (A.C.); giulia.manguso@gmail.com (G.M.); serena_valentini@yahoo.it (S.V.); susanna.sciomer@uniroma1.it (S.S.); francesco.ciciarello@uniroma1.it (F.C.); giulia.benedetti@uniroma1.it (G.B.); francesco.fedele@uniroma1.it (F.F.); dario.vizza@uniroma1.it (C.D.V.); 2 Internal Medicine Department, Ospedale S. Pertini, 00157 Rome, Italy; nadiacedrone@gmail.com

**Keywords:** pulmonary embolism, chronic thromboembolic pulmonary hypertension, arterial stiffness, cardio ankle vascular index

## Abstract

Chronic thromboembolic pulmonary hypertension (CTEPH) is a severe and under-recognized complication of acute pulmonary embolism (PE). Forty consecutive patients with acute PE (Group 1), predominantly female (22, 55%) with a mean age of 69 ± 15 years, were matched for demographic data with 40 healthy subjects (Group 2), 40 systemic hypertension patients (Group 3) and 45 prevalent idiopathic pulmonary arterial hypertension (IPAH) patients (Group 4). The baseline evaluation included physical examination, NYHA/WHO functional class, right heart catheterization (RHC) limited to IPAH patients, echocardiographic assessment and systemic arterial stiffness measurement by cardio-ankle vascular index (CAVI). Patients with PE underwent an echocardiographic evaluation within 1 month from hospital discharge (median 27 days; IQR 21–30) to assess the echo-derived probability of PH. The CAVI values were significantly higher in the PE and IPAH groups compared with the others (Group 1 vs. Group 2, *p* < 0.001; Group 1 vs. Group 3, *p* < 0.001; Group 1 vs. Group 4, *p* = ns; Group 4 vs. Group 2, *p* < 0.001; Group 4 vs. Group 3, *p* < 0.001; Group 2 vs. Group 3, *p* = ns). The predicted probability of echocardiography-derived high-risk criteria of PH increases for any unit increase of CAVI (OR 9.0; C.I.3.9–20.5; *p* = 0.0001). The PE patients with CAVI ≥ 9.0 at the time of hospital discharge presented an increased probability of PH. This study highlights a possible positive predictive role of CAVI as an early marker for the development of CTEPH.

## 1. Introduction

Chronic thromboembolic pulmonary hypertension (CTEPH) is a rare and severe complication of acute pulmonary embolism (PE) [1,2,3].

The precise pathogenesis of CTEPH remains unclear, but it appears to be caused by organized intraluminal fibrothrombotic material combined with progressive microvascular remodeling. The most supported hypothesis is that local and systemic vascular mediators could be triggering the development of microvascular remodeling in CTEPH.

Progressive arteriopathy in a proximal and small pulmonary vessel leads to an increase in pulmonary vascular resistance (PVR), resulting in pulmonary arterial pressure elevation, right ventricular failure and ultimately death [4].

The disease is under-recognized due to non-specific symptoms, and the diagnosis is usually made in advanced stages of the disease, which is associated with undermined response to treatment.

Multiple risk factors are associated with CTEPH, including demographic features and clinical conditions, such as younger age, splenectomy, the elevated prevalence of inflammatory disease, myeloproliferative syndromes, non-0 type blood group, ventriculoatrial shunt, underlying autoimmune or hematological disorders (elevated Factor VIII, lupus anticoagulant or antiphospholipid syndrome) and cancer [5,6,7,8,9,10,11]. Potentially associated aetiologies were attributed to unprovoked pulmonary embolism (PE), recurrent thromboembolic events and incomplete thrombus resolution [12]. Furthermore, echocardiographic-determined pulmonary artery systolic pressure above 50 mmHg, at initial presentation, was associated with a higher risk of developing pulmonary hypertension and should trigger a high suspicion of CTEPH development at the time of hospital discharge [13,14,15,16]. Recent international guidelines recommend a close follow-up of patients with risk factors or associated conditions for CTEPH [17].

The aim of the present study was to assess whether, in patients with PE, a non-invasive approach for the measurement of arterial stiffness [18], such as the cardio-ankle vascular index (CAVI), could be useful as a marker of pulmonary and systemic vascular remodeling to highlight those patients at higher risk of CTEPH development. This would allow for an early marker in the pathogenesis of CTEPH.

## 2. Materials and Methods

### 2.1. Study Population

The study population included 40 consecutive patients with acute PE referred to our hospital between 1 June and 31 December 2019. Subjects in this group (Group 1) were matched for demographic data with 40 healthy subjects (Group 2), 40 systemic hypertension patients (Group 3), and 45 prevalent idiopathic pulmonary arterial hypertension patients (Group 4), recruited from our local database.

The diagnosis of PE was confirmed by diagnostic algorithm including clinical presentation, computed tomographic pulmonary angiography and lung scintigraphy, according to the ESC/ERS guidelines [17]. Hemodynamic instability is defined as one of the following clinical manifestations at presentation: cardiac arrest; obstructive shock (with systolic BP < 90 mmHg or vasopressors required to achieve a BP ≥ 90 mmHg); persistent hypotension (with systolic BP < 90 mmHg or systolic BP drop ≥40 mmHg, lasting longer than 15 min and not caused by new-onset arrhythmia, hypovolaemia, or sepsis as a summarized in international guidelines/according to predefined criteria [17].

The diagnostic work-up of IPAH was established according to the ESC/ERS guidelines with the typical hemodynamic profile of precapillary pulmonary hypertension (defined by a mean pulmonary artery pressure, mPAP ≥ 25 mmHg, a pulmonary artery wedge pressure, PAWP ≤ 15 mmHg, and a pulmonary vascular resistance, PVR ≥ 3 Wood Units) and a diagnostic algorithm including pulmonary function tests, perfusion lung scan, computer tomography scan and echocardiography [19].

The diagnosis of systemic hypertension was defined as a persistent elevation of systolic blood pressure (SBP) ≥ 140 mmHg and/or diastolic blood pressure (DBP) ≥ 90 mmHg according to the ESC/ESH guidelines definition criteria [20]. Patients data were safely tracked through the local database used for the prospective follow-up of IPAH patients [21]. Patients for the matched groups were derived from historical cohorts followed at our center.

The baseline evaluation included medical history, physical examination, NYHA/WHO functional class, right heart catheterization (RHC; limited to IPAH patients), and peripheral artery stiffness measurement by cardio ankle vascular index (CAVI). In the PE cohort, the CAVI was measured in all patients at the time of hospital discharge. As part of common clinical practice, patients with PE underwent an echocardiographic evaluation within 1 month from hospital discharge.

All patients were included in the study after informed consent. The study protocol was approved by the Institutional Review Board for human studies of the Policlinico Umberto I—Sapienza University of Rome (Reference N. 1561/14).

### 2.2. Right Heart Catheterization

A hemodynamic evaluation was made with a standard technique. Pressures were measured from the mid-chest position, in the supine position, with a fluid-filled catheter and pressure transducer, recording the average values over three respiratory cycles, as previously described by our group [22,23]. Cardiac output (CO) was measured by the thermodilution technique (American Edwards Laboratories, Santa Ana, CA, USA), and pulmonary vascular resistance (PVR) was calculated with the formula PVR = (mPAP−PWP)/CO.

### 2.3. Echocardiographic Assessment

The echocardiographic evaluation was performed in accordance with the American Society of Echocardiography Guidelines [24]. All echocardiographic data were acquired by dedicated operators, with the patient in the left lateral decubitus position using commercially available equipment (Vivid S6, GE). The peak pulmonary artery systolic pressure (PASP) was estimated by adding the right atrial pressure (RAP; estimated by the diameter and collapsibility of inferior vena cava) to the systolic transtricuspid pressure gradient, measured according to the simplified Bernoulli’s equation [25].

Left ventricle (LV) and right ventricle (RV) end-diastolic diameter, inter-ventricular septum thickness and left atrium size were measured in all the patients. LV ejection fraction (LVEF) was calculated using the biplane modified Simpson’s rule.

In addition, the following echocardiographic signs of pulmonary arterial hypertension (PAH) were evaluated to assess the probability of PAH in accordance with the ESC/ERS guidelines: RV/LV basal diameter ratio, left ventricular eccentricity index in systole, RV outflow Doppler acceleration time, pulmonary artery diameter, diameter and respiratory variation of the inferior vena cava, and right atrial area. Patients were considered at high probability of PAH if peak tricuspid regurgitation velocity was >3.4 m/s without other PAH signs or if peak tricuspid regurgitation velocity was between 2.9 and 3.4 m/s associated with PAH signs.

Intraobserver and interobserver variability for echocardiographic measurements was previously reported by our group [26,27,28,29,30].

### 2.4. Cardio-Ankle Vascular Index (CAVI)

The CAVI represents a non-invasive approach for the direct measurement of arterial stiffness. It is a blood pressure-independent index that measures the stiffness of the aorta, femoral artery and tibial artery. It was shown to have a better reproducibility for clinical use [18]. It was also reported that CAVI correlates with other cardiovascular risk markers, such as intimal-medial thickening and coronary atherosclerosis [31,32]. It is calculated using the underlying algorithm enriched with measurements from an electrocardiogram, phonocardiogram, brachial artery waveform, and ankle artery waveform [17]: CAVI = α {(2þ/∆P) × ln (SBP/DBP) PWV^2^} +β, where ∆P is SBP − DBP, þ is blood density, PWV is pulse wave velocity and α and β are constants.

Scale conversions constants are determined so as to match CAVI with PWV using the Hasegawa method. All measurements and calculations are made together and automatically in Va-Sera (Fukuda Denshi Co. Ltd., Tokyo, Japan). This equation was derived from Bramwell–Hill equation and the stiffness parameter β. CAVI reflects the stiffness of the aorta, femoral artery and tibial artery as a whole and is theoretically not affected by blood pressure [18]. This device utilizes blood pressure cuffs with sensors on all four limbs to generate plethysmographs. Patients are tested for CAVI and ankle-brachial index (ABI) on the same day. The cuffs are placed on bilateral upper and lower extremities while the patient is in a supine position with the limbs at the same level as the heart, in a comfortable position in a warm room. Measurement of ABI is performed during CAVI measurement.

### 2.5. Statistical Analysis

To compensate for the lack of randomization methods, the nearest neighbor matching method 1:1, by the exact distance, was used to balance the distribution of covariates in the groups, diagnosing the quality of the resulting matching through the standardized difference in means (the difference in means of each covariate divided by the standard deviation in the fully treated group). This method was chosen as the most effective method (increased power and decreased bias) for small group sizes [33].

Continuous data are expressed as mean ± standard deviation, while not normally distributed variables are reported as medians and interquartile ranges (IQR). Categorical data are expressed as counts and proportions.

The comparisons among groups were performed with a 2-way analysis of variance (ANOVA) for variables normally distributed. If significant differences were found, posthoc comparisons (Duncan’s multiple range test or Scheffé test) were used to determine the statistical significance among groups. The Kruskal–Wallis test was used for non-parametric comparison. Categorical data were analyzed with the chi-square or Fisher’s exact test.

A linear regression analysis was performed to assess the relationships between CAVI and PASP and expressed as a Pearson’s correlation coefficient. The predicted probability of high-risk criteria for PH based on CAVI was assessed by logistic analysis.

Sensitivity (Se), specificity (Sp), negative predictive value (NPV) and positive predictive value (PPV) were calculated for the prediction of CTEPH.

All statistical analyses were performed using IBM SPSS Statistics for Windows software (version 25.0; IBM Corp., Armonk, NY, USA). All statistical tests were two-sided, and a *p*-value < 0.05 was considered statistically significant.

## 3. Results

Anthropometric, clinical and echocardiographic data of the study population are summarized in Table 1.

Patients with PE (Group 1) were predominantly female (22, 55%) with a mean age of 69 ± 15 years. The majority of patients were in NYHA/WHO functional class II and III (13 (32.5%) and 16 (40%), respectively). In our cohort, 90% of patients were on stable hemodynamic conditions at the time of PE diagnosis: 22 (55%) patients were classified as intermediate risk and 14 (35%) patients were classified as low-risk of early death. Four patients (10%) were hemodynamically unstable at presentation: three had persistent hypotension and one cardiogenic shock. Among risk factors for PE, immobilization >3 days, previous venous thromboembolism and history of cancer were present in 10 (25%), 9 (22.5%), and 6 (15%) patients, respectively. The cohort included a low percentage of patients with estroprogestin therapy (4%) and autoimmune and hematological disorders (2%).

**Figure 1:**
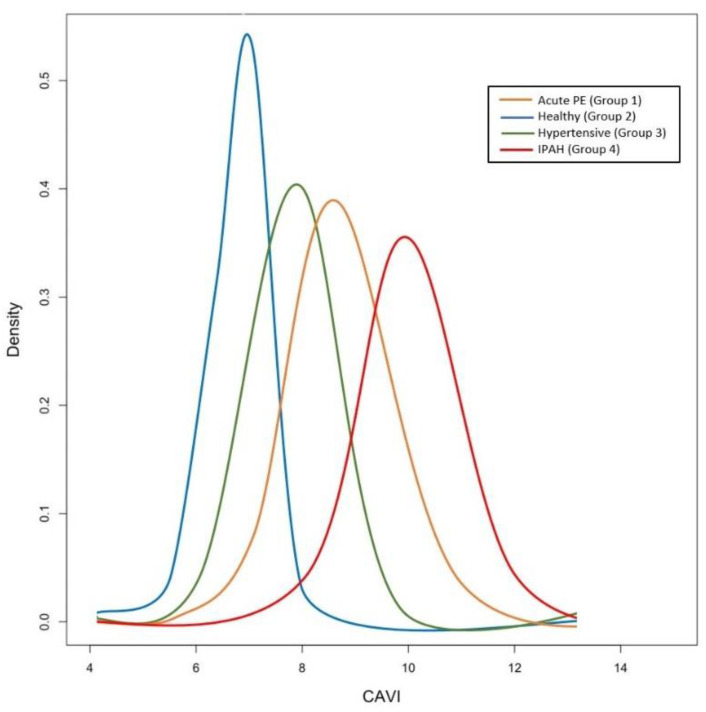
Distribution of the CAVI values for each cohort of patients. **Group 1:** patients with acute PE (orange line); **Group 2:** healthy subjects (blue line); **Group 3:** patients with systemic hypertension (green line); **Group 4:** patients with idiopathic pulmonary arterial hypertension (red line). CAVI was significantly increased in PAH and in acute pulmonary embolism patients compared with healthy subjects and systemic hypertension patients. Abbreviation: CAVI: cardio-ankle vascular index; PE: pulmonary embolism; IPAH: idiopathic pulmonary arterial hypertension.

In this cohort of patients, the CAVI measured at the time of hospital discharge showed a median value of 8.6 (IQR 7.8; 9.0; Figure 1). The echocardiographic evaluation within 1 month (median 27 days; IQR 21–30) from hospital discharge showed normal LV size and systolic function in the majority of the patients. Increased RV size was detected in five patients, and decreased tricuspid annular plane systolic excursion (TAPSE) was found in three patients (Table 1). The PASP was estimated at 29.4 ± 5.6 mmHg. Five patients (12.5%) were found at high probability of PH.

The healthy matched group (Group 2) had a normal echocardiographic assessment of left and right heart metrics. The CAVI assessment showed a median value of 6.9 (IQR 6.6; 7.7; Figure 1).

The hypertensive matched group (Group 3) was characterized by increased left atrial size associated with left ventricular hypertrophy and ejection fraction within the normal range. The SBP and DBP were significantly higher in these patients compared with the other cohorts (Group 3 vs. Group 1, *p* < 0.01; Group 3 vs. Group 2, *p* < 0.001; Group 3 vs. Group 4, *p* < 0.001). Among hypertensive patients, the CAVI assessment showed a median value of 7.9 (IQR 6.8; 8.5; Figure 1).

In the IPAH cohort (Group 4), 13 (28.9%) patients had mPAP 25–34 mmHg, 22 (48.9%) patients had mPAP 35–44 mmHg and 10 (22.2%) patients had mPAP ≥ 45 mmHg. Five (11.1%) patients were WHO functional class I, 18 (40%) patients were WHO functional class II, 18 (40%) WHO functional class III and 4 (8.9%) WHO functional class IV. In this group, CAVI showed a median value of 9.9 (IQR 9.2; 10.7; Figure 1).

**Figure 2:**
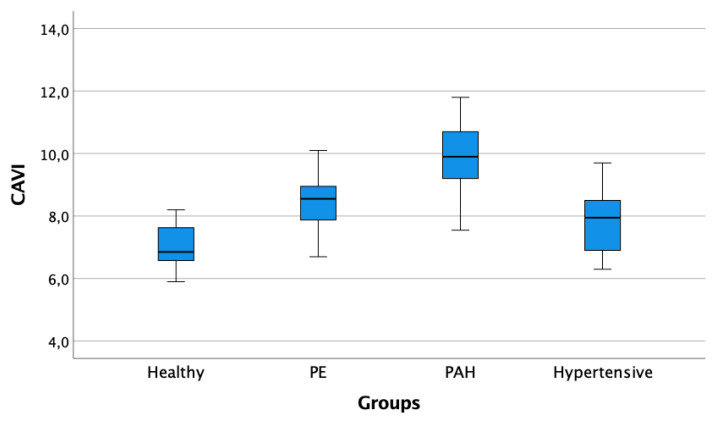
Boxplots of CAVI values among the different cohorts of patients. Box edges represent the 25th (Q1) and 75th (Q3) quantiles, respectively. The upper whisker is drawn at the greatest value smaller than 1.5 IQR above the third quartile, while the lower whisker is drawn at the smallest value greater than 1.5 IQR below the first quartile. Abbreviations: CAVI: cardio-ankle vascular index; PE: pulmonary embolism; PAH: pulmonary arterial hypertension.

The CAVI were significantly higher in the PE and IPAH groups compared with the others (Group 1 vs. Group 2, *p* < 0.001; Group 1 vs. Group 3, *p* < 0.001; Group 1 vs. Group 4, *p* = ns; Group 4 vs. Group 2, *p* < 0.001; Group 4 vs. Group 3, *p* < 0.001; Group 2 vs. Group 3, *p* = ns; Figure 2).

**Figure 3:**
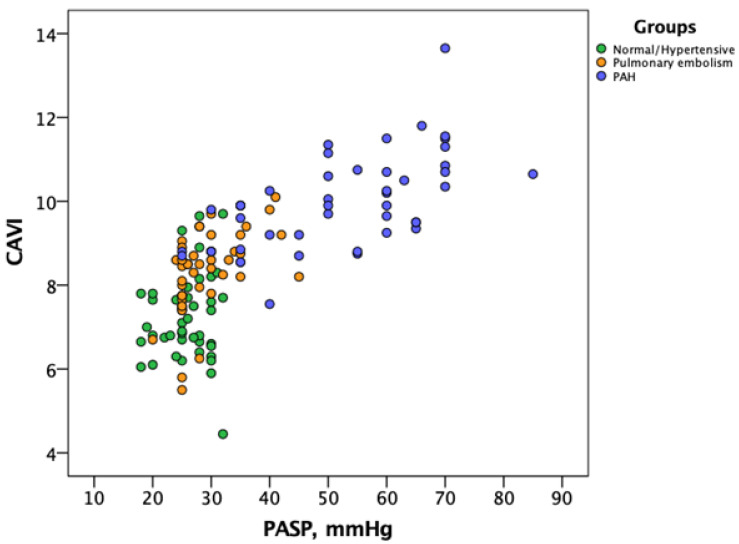
Correlation between CAVI (Y-axis) and echo-derived PASP (X-axis) based on patients cohort. The 4 groups of patients are reported in the same scatterplot. Group 1 (patients with acute pulmonary embolism): orange circles; Group 2 and Group 3 (healthy subjects and patients with systemic hypertension): green circles; Group 4 (patients with idiopathic pulmonary arterial hypertension): blue circles. Patients with pulmonary embolism and idiopathic pulmonary arterial hypertension present higher values of CAVI compared with normal and hypertensive patients. Abbreviations: CAVI: cardio-ankle vascular index; PASP: pulmonary arterial systolic pressure.

Interestingly, CAVI were positively correlated with the PASP values measured by echocardiography at 1-month assessment (r^2^ = 0.55, *p* = 0.0001). Figure 3 shows the distribution of CAVI versus PASP values among the different cohorts of patients, with PE and IPAH patients presenting higher values of CAVI compared with normal and hypertensive patients.

**Figure 4:**
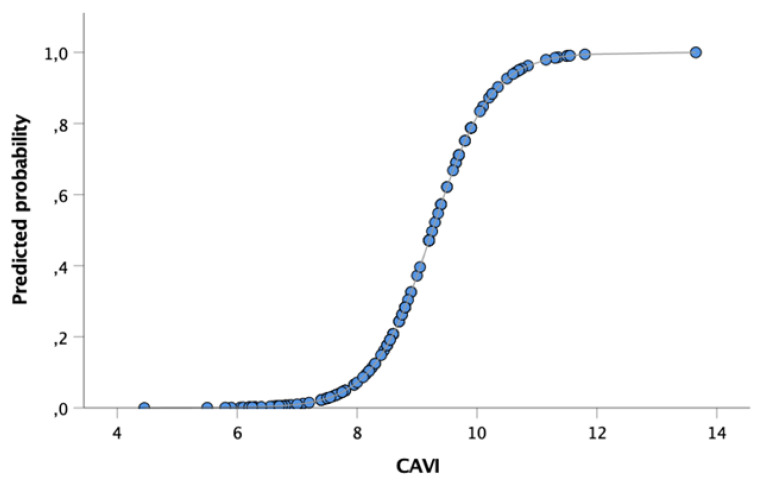
Predicted probability of high-risk echocardiography criteria of PAH at second observation (within 1 month from diagnosis) based on the CAVI value at PE diagnosis. Abbreviations: CAVI: cardio-ankle vascular index.

The predicted probability of echocardiography-derived high risk of PH increases for any unit increase of CAVI (OR 9.0; C.I. 3.9–20.5; *p* = 0.0001). As shown in Figure 4, the higher quartile of PE patients based on the CAVI (≥9.0) at the time of hospital discharge presented an increased probability of PH (≥60%) at the first echocardiographic evaluation within 1 month.

In our study, among patients found to be with a high echocardiographic probability of PAH, CTEPH was confirmed by right heart catheterization in four patients after 3 months of anticoagulation therapy (Table 2).

The Se, Sp, NPV and PPV of CAVI for the prediction of CTEPH were 100%, 91%, 100% and 63%, respectively.

## 4. Discussion

Our results showed increased arterial stiffness measured by cardio-ankle vascular index (CAVI) in a significant proportion of patients with acute pulmonary embolism. Additionally, increased arterial stiffness is associated with a high probability of echocardiographic signs of PAH at short-term follow-up.

The demographic characteristics and the distribution of risk factors and the associated condition for PE of our study population were in agreement with the Italian Pulmonary Embolism Registry (IPER) [34]. Indeed, in the IPER, the mean age was 69 ± 15 years, and the majority of patients were females. The majority of patients were hemodynamically stable at the time of diagnosis.

The CAVI is a new non-invasive parameter that was developed for the evaluation of arterial stiffness [18,35]. It is acknowledged in the American Heart Association’s scientific statement for improving and standardizing vascular research [36]. Comparing with the pulse wave velocity (PWV), the CAVI is easier to acquire and less affected by blood pressure [32,37,38].

In our study, patients with acute PE showed significantly increased echo-derived arterial stiffness compared with normal subjects and hypertensive patients, as already reported in PAH patients [39]. The increased CAVI found in our PE cohort was not statistically different from our IPAH cohort, although a trend to higher values was observed in the latter population. Increased arterial stiffness by PWV was also reported in a small cohort of CTEPH patients compared with healthy subjects (10.3 ± 2.5 m/s vs. 9 ± 1.3 m/s, *p* < 0.05) [40].

Interestingly, it is widely accepted that in systemic circulation, several factors may ultimately lead to increased arterial stiffness, as increased age, gender, hypertension and other risk factors of cardiovascular diseases [41,42,43,44]. Additionally, it was demonstrated that arterial stiffness is related to systemic inflammation [45], known to be activated in acute pulmonary embolism [46,47,48]. Indeed, there is a growing interest in the role of inflammation in the pathogenesis and progression of CTEPH. Zabini D et al. found a significant dysregulation of circulating inflammatory cytokines in both CTEPH and IPAH patients, showing increased expression of a broad range of inflammatory factors, such as IL-6, IL-8, IL-10, y-interferon-induced protein 10 (IP-10), monocyte chemotactic protein-1 (MCP-1) and macrophage inflammatory protein-9 [49]. Such inflammatory activation was further described in IPAH [50,51,52,53,54,55,56,57].

Therefore, it is not surprising that in the present study, a proportion of patients with recent PE showed increased arterial stiffness, suggesting systemic action of locally produced inflammatory mediators in the pulmonary circulation. Such pathophysiologic hypothesis should be further investigated, as blood/tissue samples were not considered in the present study.

To our knowledge, this is the first application of CAVI in patients with acute PE. These results provide interesting information regarding the association between increased arterial stiffness and high-risk probability of PH at echocardiographic evaluation within 1 month from hospital discharge. CAVI values ≥9.0 showed an increased probability of high-risk signs of PH at the first echocardiographic assessment after acute PE. Of note, 25% of patients with acute PE had CAVI values ≥9.0, and 10% values >9. A significant correlation between CAVI-derived arterial stiffness metrics and echo-derived pulmonary systolic pressure estimates was already observed in patients with systemic inflammatory diseases as scleroderma [58].

As it is recognized that the increased PVR in CTEPH is caused by obstruction of pulmonary arterial vessels by organized thromboemboli and by vascular remodeling of small unobstructed vessels [59], the increased CAVI score observed in a significant proportion of patients with acute PE may reflect the complex interaction between autocrine, paracrine and systemic hormonal and inflammatory factors shown in acute PE [46,47,48], leading to an increased probability of CTEPH development. Among PE patients, 10% of those with CAVI values >9 were confirmed with CTEPH diagnosis, allowing high PPV (63%) without losing the NPV (100%). Figure 4 explores the tight association between the CAVI score and the probability of PH at echocardiographic assessment within 1 month from the acute event.

In this perspective, the assessment of CAVI in the subset of patients with acute PE could improve risk stratification for the development of CTEPH.

Indeed, current risk assessment tools are characterized by high negative predictive value but low positive predictive value, allowing to identify those patients with previous pulmonary embolism at low risk of developing CTEPH [60]. Klok FA et al. found 99.6% NPV, but 10% PPV, for an overall score ≤6, including unprovoked PE, known hypothyroidism, symptom onset >2 weeks before PE diagnosis, right ventricular dysfunction on CT or echocardiography, known diabetes mellitus, thrombolytic therapy or embolectomy.

While early diagnosis remains a challenge in CTEPH and current risk assessment largely disregards disease mechanisms and relies on clinical presentation, risk factors and associated conditions, our approach highlights potential neurohormonal-induced changes in the systemic circulation reflecting pulmonary vascular changes; this is important to the pathophysiology of CTEPH, and may pave the way for further investigation within and between the current approach. Identifying increased arterial stiffness as a reflection of pulmonary vascular remodeling induced by PE seems to improve the positive predictive value of risk assessment for CTEPH development.

## 5. Conclusions

CAVI is increased in patients with acute pulmonary embolism compared with healthy subjects and systemic arterial hypertension.

Patients with CAVI (≥9.0) at the time of hospital discharge presented a higher likelihood of high-risk criteria of PH at first echocardiographic evaluation, highlighting a possible positive predictive role of CAVI as an early marker for the development of CTEPH.

Further studies, including larger numbers of patients, are needed to validate the association between CAVI and CTEPH development.

## 6. Limitations

The present study has limitations. This is a single-center study with small sample size. Second, patients with PE have several comorbidities potentially affecting arterial stiffness, and this can contribute to reducing the accuracy of CAVI as an index of systemic hormonal activation induced by pulmonary embolism. However, the rate of comorbidities between the PE and the hypertensive group was not statistically different.

As CAVI and echocardiographic measurements before patients’ acute PE are not available, we cannot exclude pre-existing vascular damage or pre-existing PH.

Furthermore, lower CAVI may be observed in younger IPAH patients with less advanced disease [61].

Further studies with larger cohorts of patients are needed to confirm our results.

## Figures and Tables

**Table 1:** Baseline demographic and clinical characteristics of the study population.

	Acute PE	Healthy	Hypertensive	IPAH
**Patients, *n***	40	40	40	45
**Age, years**	69 ± 15	67 ± 12	69 ± 13	67 ± 11
**Gender, F:M, *n***	22:18	21:19	20:20	23:22
**Height, cm**	163 ± 9	165 ± 8	164 ± 9	163 ± 10
**Weight, Kg**	68 ± 12	66 ± 10	70 ± 11	68 ± 9
**Comorbidities, *n* (%)**				
Smoking	7 (17.5%)	6 (15%)	8 (20%)	0 (0%)
Hypercholesterolemia	10 (25%)	4 (10%)	11 (27.5%)	6 (13.3%)
Diabetes mellitus	6 (15%)	0 (0%)	5 (12.5%)	4 (8.8%)
systemic hypertension	12 (30%)	0 (0%)	40 (100%)	0 (0%)
**Risk factors for PE, *n* (%)**				
immobilization >3 days, %	10 (25%)	0 (0%)	0 (0%)	0 (0%)
previous VTE, %	9 (22.5%)	0 (0%)	0 (0%)	0 (0%)
history of cancer	6 (15%)	0 (0%)	0 (0%)	0 (0%)
**NYHA/WHO, *n* (%)**	2.6 ± 0.6	1.0	1.5 ± 0.7	2.5 ± 1.4
I	7 (17.5%)	40 (100%)	32 (80%)	5 (11.1%)
II	13 (32.5%)	0 (0%)	8 (20%)	18 (40%)
III	16 (40%)	0 (0%)	0 (0%)	18 (40%)
IV	4 (10%)	0 (0%)	0 (0%)	4 (8.9%)
**SBP, mmHg**	117 ± 18	115 ± 12	140 ± 14	110 ± 13
**DBP, mmHg**	71 ± 9	66 ± 7	89 ± 8	68 ± 8
**HR, b/m**	84 ± 13	79 ± 6	80 ± 7	80 ± 11
**CAVI**	8.6 (7.8; 9.0)	6.9 (6.6; 7.7)	7.9 (6.8; 8.5)	9.9 (9.2; 10.7)
Echocardiography *				
LVEDd (mm)	48.2 ± 4.3	47.8 ± 3.8	49.1 ± 4.3	43.5 ± 4.3
LVEDA (cm^2^)	38.8 ± 2.5	40.1 ± 4.8	42.1 ± 5.8	30.8 ± 4.7
LVESA (cm^2^)	19.4 ± 4.4	20.3 ± 4.3	21.5 ± 5.2	17.6 ± 4.4
LA area, (cm^2^)	18.2 ± 3.1	15.2 ± 2.4	21.5 ± 3.2	13.6 ± 3.4
LVEF, %	56 ± 5	58 ± 4	57 ± 4	56 ± 4
TAPSE, mm	20.2 ± 3.3	24.5 ± 2.8	25.1 ± 2.7	16.9 ± 3.2
RA area, cm^2^	20.1 ± 2.8	13.3 ± 2.5	14.1 ± 2.7	26.2 ± 6.6
PASP, mmHg, *n* (%)				
≥40	8 (20%)	0 (0%)	0 (0%)	45 (100%)
RV/LV ratio, *n* (%)				
1	2 (5%)	0 (0%)	0 (0%)	18 (40%)
>1	3 (7.5%)	0 (0%)	0 (0%)	27 (60%)
<1	35 (87.5%)	40 (100%)	40 (100%)	0 (0%)

* The echocardiographic assessment within 1 month from hospital discharge is reported in the same table. LEGEND: PE: pulmonary embolism; CTEPH: Chronic thromboembolic pulmonary hypertension; VTE: Venous thromboembolism; WHO: World Health Organization; SBP: systolic blood pressure; DBP: diastolic blood pressure; HR: heart rate; CAVI: cardio-ankle vascular index (values are reported as median and inter-quartile range); LVEDd: left ventricular end-diastolic dimension; LVEDA: left ventricular end-diastolic area; LVESA: left ventricular end-systolic area; LA: left atrial; LVEF: left ventricular ejection fraction; TAPSE: tricuspid annular plane systolic excursion; RA area: right atrium area; PASP: pulmonary artery systolic pressure; RV: right ventricular; LV: left ventricular.

**Table 2:** Baseline demographic and clinical characteristics of patients with acute pulmonary embolism and CTEPH.

	Acute PE	CTEPH	*p*
**Patients, *n***	40	4	
**Age, years**	69 ± 15	70 ± 4.2	ns
**Gender, F:M, *n***	22:18	2:2	ns
**Height, cm**	163 ± 9	162 ± 8	ns
**Weight, Kg**	68 ± 12	71 ± 5.6	ns
**WHO, *n* (%)**	2.6 ± 0.6	2.5 ± 0.7	ns
I	7 (17.5%)	0 (0%)	
II	13 (32.5%)	1 (25%)	
III	16 (40%)	3 (75%)	
IV	4 (10%)	0 (0%)	
**CAVI**	8.6 (7.8; 9.0)	9.75 (9.4; 10.1)	<0.01
Echocardiography *			
TAPSE, mm	20 ± 3	17.5 ± 3.5	<0.01
RA area, cm^2^	20 ± 2.8	23.5 ± 3.5	<0.01
PASP, mmHg, *n* (%)			
≥40	8 (20%)	4 (100%)	<0.01
RV/LV ratio, *n* (%)			<0.01
1	2 (5%)	1 (25%)	
>1	3 (7.5%)	3 (75%)	
<1	35 (87.5%)	0 (0%)	

* The echocardiographic assessment within 1 month from hospital discharge is reported in the same table. LEGEND: PE: pulmonary embolism; CTEPH: Chronic thromboembolic pulmonary hypertension; VTE: Venous thromboembolism; WHO: World Health Organization; SBP: systolic blood pressure; DBP: diastolic blood pressure; HR: heart rate; CAVI: cardio-ankle vascular index (values are reported as median and inter-quartile range); LVEDd: left ventricular end-diastolic dimension; LVEDA: left ventricular end-diastolic area; LVESA: left ventricular end-systolic area; LA: left atrial; LVEF: left ventricular ejection fraction; TAPSE: tricuspid annular plane systolic excursion; RA area: right atrium area; PASP: pulmonary artery systolic pressure; RV: right ventricular; LV: left ventricular.

## Data Availability

Due to privacy and ethical concerns, the data cannot be made available. However, a specific request might be considered by the authors.

## Abbreviations

ABI	ankle-brachial index
CAVI	cardio ankle vascular index
CO	Cardiac output
CTEPH	Chronic thromboembolic pulmonary hypertension
DBP	diastolic blood pressure
IP-10	y-interferon-induced protein
PAWP	pulmonary artery wedge pressure
IPAH	idiopathic pulmonary arterial hypertension
LV	left ventricle
PE	Pulmonary embolism
LVEF	left ventricle ejection fraction
MCP-1	monocyte chemotactic protein-1
mPAP	mean pulmonary artery pressure
PH	Pulmonary Hypertension
NYHA/WHO	New York Heart Association/World Health Organization
PA	pulmonary arterial
PAH	pulmonary arterial hypertension
PASP	pulmonary artery systolic pressure
PVR	pulmonary vascular resistance
PWV	pulse wave velocity
RAP	right atrial pressure
RHC	right heart catheterization
RV	right ventricle
SBP	systolic blood pressure
TAPSE	Tricuspid annular plane systolic excursion
